# A Comparative Analysis of Sustainable Design Tools for Product Redesign Within a Business Context

**DOI:** 10.3390/biomimetics10100667

**Published:** 2025-10-03

**Authors:** Sarah McInerney, Peter H. Niewiarowski

**Affiliations:** 1 The Biomimicry Institute, 1800 South 22nd Avenue, Suite 120, Box 656, Bozeman, MT 59718, USA; sarah.mcinerney@biomimicry.org; 2 Integrated Bioscience, Department of Biology, University of Akron, Akron, OH 44325, USA

**Keywords:** sustainable innovation, biomimicry, sustainable design tools, business context

## Abstract

In recent years, corporate perceptions of environmental sustainability have shifted from viewing it as a compliance burden to recognizing it as a strategic driver of innovation and competitive advantage, prompting a demand for effective sustainable design tools. Traditionally, tools like the Life Cycle Assessment (LCA) tool have been used to evaluate environmental impacts, yet their complexity, cost, and retrospective focus make them impractical for driving early-stage, disruptive innovation. Although biomimicry has emerged as a promising approach, adopting this novel interdisciplinary design practice within a corporate setting requires significant resources and time, disrupting established processes. Therefore, the biomimicry Life Principles (LPs) tool, a guiding sustainable design tool of the practice, provides an opportunity to lower the barrier to the entry of biomimicry within a corporate setting and potentially increases adoption of the broader practice. This comparative study seeks to explore the creative potential, and practical value of the biomimicry LPs tool compared to the traditional LCA approach while exploring the intrinsic motivation of R&D practitioners to implement these tools within a virtual product redesign workshop. To derive our conclusions, we employed a mixed-methods approach comprising a 23-item survey designed to assess practitioners’ intrinsic motivation and perceived practical value of the implemented tool alongside an external evaluation of the creativity of all generated design concepts. Together, these methods provide empirical evidence of the biomimicry LPs tool’s potential to enhance creative output, require minimal adoption effort, and act as a catalyst for whole-systems thinking in sustainable innovation. These findings offer compelling evidence to support their strategic addition to existing R&D toolkits and workflows. By highlighting the efficacy, accessibility, and intrinsic motivation of R&D professionals to use biomimicry LPs, the results underscore the viability of this tool to streamline the integration of biomimicry design thinking into real-world workflows. As such, they represent a pragmatic and scalable pathway to catalyze broader and deeper engagement with biomimicry across corporate contexts.

## 1. Introduction

Over the past decade, perceptions of environmental sustainability in business have shifted from being viewed as a costly obligation to a source of innovation and competitive advantage [1]. Increasingly, stakeholders—including customers, employees, suppliers, and investors—are holding companies accountable for their sustainability practices [2,3,4]. In response, sustainable product development has gained prominence, highlighting both the demand for new design tools and the growth of related academic literature [5,6,7]. Traditionally, sustainable design tools have been applied at the end of the design process to evaluate environmental performance; however, as 70–80% of key design decisions occur at the front end of the design process, integrating these tools at the front end of innovation offers greater potential for embedding sustainability into product development [1].

The front end of innovation is resource-intensive, requiring several iterations of problem exploration, brainstorming, and ideation prior to product development. To streamline this process, organizations often turn to data-driven reductionist tools such as Life Cycle Assessment (LCA), an evaluative tool that meticulously quantifies the environmental impact of a product’s life cycle across a diversity of impact categories, such as abiotic depletion potential, acidification potential, eutrophication potential, global warming potential, and others. Results of LCAs normally take the form of technical reports that identify “hotspots” of negative environmental performance that can be used to target specific areas to improve the efficiency or sustainability of a product or process across its life cycle [8,9]. While LCA tools provide excellent insight into incremental change, they offer limited support for truly disruptive innovation, which could have a larger impact on sustainability [8,9]. Furthermore, the extensive quantitative analysis required to produce an LCA report presents significant challenges for industry practitioners. Therefore, the degree of implementation within a corporate environment varies from completing internal reports, referencing competitors, or minimizing the categories of consideration, such as focusing solely on carbon or water implications [10,11,12]. This approach can lead to shortcomings with rigor and accuracy during implementation [10,13,14]. These constraints highlight the importance of design tools that not only provide useful environmental insights but also foster intrinsic motivation among practitioners [15]. Intrinsic motivation is the desire to perform an action because of the interest, enjoyment, and satisfaction derived from the action itself, rather than external rewards. It is directly related to self-determination theory, which posits that those who are internally motivated following task participation are more likely to internalize the task and excel at implementation [16]. Given the budgetary and timeline constraints of corporate innovation practices, this intrinsic motivation is critical to ensure practitioner commitment to environmentally sustainable innovation, prioritizing decisions at the front end of the design process that lead to a positive environmental impact.

One alternative approach that has gained traction for sustainable innovation is biomimicry [1,6,17,18,19,20,21,22,23,24,25,26,27]. Biomimicry involves the conscious emulation of biological forms, processes, and systems to inform sustainable innovation. However, realizing innovative environmentally sustainable solutions requires a design process that exceeds the simplified mimicry or emulation of function alone. It requires an ethical commitment and recognition of humanity’s interdependence with nature [28,29]. While biomimetic practice often focuses solely on emulation, neglecting the ethical and relational dimensions, this limited approach risks producing shallow solutions that fail to deliver environmentally sustainable solutions—termed the “biomimetic promise”—without the practitioner’s conscious commitment to an ethos of respect and ecological integration in decision making [26,30]. To ensure a deeper level of biomimicry practice, the biomimicry life principles (LPs) tool, the guiding sustainable design tool of the practice [28], is implemented at the front end of the design process to inform concept development and at the end of the process to evaluate the design solution against project and sustainability goals. The biomimicry LPs comprise a static list of design principles grounded in the understanding that life on Earth has evolved within specific biophysical constraints, referred to as Earth’s operating conditions, and has developed strategies to thrive under these conditions—adaptation. In the context of sustainability and ecological design, these principles guide the creation of systems, products, and processes that are aligned with the planet’s natural processes, thereby promoting long-term ecological resilience and viability.

As a sustainable design tool, biomimicry LPs provide qualitative, generative prompts that stimulate discussion throughout a product’s life cycle, as opposed to the more reductionist tool, LCA, which focuses on quantifiable metrics of environmental impact. Generative approaches have been shown to foster disruptive innovation [8,9], and unlike LCA, biomimicry LPs require minimal time and budgetary resources for implementation, as they comprise a static list of principles and do not require external consultation or extensive data collection and analysis to implement [21,28,31]. This is particularly relevant in the context of industrial R&D, whose technical professionals often gravitate toward data-driven, evaluative approaches, suggesting that a tool like LCA would be more aligned with their preferences. However, despite its analytical rigor, the resource-intensive requirements of an LCA can hinder its practical implementation [10,11,12]. In contrast, biomimicry LPs, learning from nature’s efficiency, adaptability, and closed-loop systems, can inform solutions that are ecologically regenerative and resource-efficient. By adhering to these principles, human-made products and systems can emulate ecological constraints that sustain life, fostering conditions conducive to both human and planetary well-being.

Given this potential, the relationship between nature-inspired innovation (sensu [23]; including but not limited to biomimicry) and sustainability is the subject of considerable and ongoing research from diverse interdisciplinary perspectives. Indeed, Speck et al. [26] observed an exponential increase in research studies including the terms “biomimetics and sustainability,” reflecting both the rapid growth of interest in this area and the complexity of its study, given the varied definitions of sustainability and the diversity of disciplines developing biomimicry tools and methods [32]. This interdisciplinary landscape spans biology, engineering, design, and business, and has prompted the creation of tools to help navigate disciplinary boundaries [33,34]. Moreover, cross-sector collaboration introduces additional challenges, as market and economic forces shape innovation objectives and outcomes in nontrivial ways [35,36,37,38,39], leading to highly varied applications and outcomes of biomimicry in practice. Within this broader context, our study is situated in a literature that investigates sustainable design tools from multiple perspectives, including creativity, innovation, practical application, academic versus corporate settings, validity and reliability of sustainable design outcomes, resource infrastructure required for use, and cultural conditions supporting success (see Faludi et al. [40] for a review of sustainable design tools). All of these themes are directly relevant to the development, adoption, use, deployment, and outcomes of sustainable design tools, especially nature-inspired innovation (sensu Mead et al. [23]), and many have explored biomimicry in particular, including authors from fields such as design, business, biology, and engineering [5,32,34,38,39,41].

It is beyond the scope of this study to review each of these deep literatures. Instead, we explicitly situate our contribution at several of these intersections, comparing two sustainable design tools, Life Cycle Assessment (LCA) and biomimicry Life Principles (LPs), evaluating them in an authentic corporate R&D setting with respect to several qualities that we and others have argued are necessary (though not sufficient) to support progress towards sustainable innovation objectives: creative potential, intrinsic motivation, and practical value. While prior work has directly or indirectly considered such qualities for the adoption or use of LCA [41,42,43] and biomimicry tools [21,23,31,38], we believe this is the first study to directly contrast LCA and biomimicry LPs side by side in an authentic corporate setting. This comparison is especially timely given the growing demand for sustainable design tools that provide environmental value while also aligning with organizational constraints—requiring minimal training, budget, and workflow disruption [31,38,44]. To date, the literature for successful implementation within this context has suggested that an initial low barrier to entry and clear return on investment is essential [21,44]. This includes flexible independent design tools that can be integrated into existing workflows without requiring extensive training or external collaborations and that do not disrupt established deadlines or budget constraints as to garnering leadership support [38,44]. To garner practitioners’ justification to adopt a new tool, there must be additional unique value beyond their current streamlined design processes. With the highest potential for sustainable innovation being at the front end of innovation, such additional value may include advancing the creativity of solutions and being a tool that is intrinsically motivating to use or can foster a whole-systems thinking approach [35].

The objective of this study is to compare the creative potential, intrinsic motivation, and practical value of two sustainable design tools—a simplified LCA report and the biomimicry LPs tool—as applied by corporate R&D practitioners within a corporate setting to inform the early stages of a consumer product redesign. By doing so, we aim to advance a cross-disciplinary understanding of how sustainable design tools contribute to innovation and to encourage further research, bridging sustainability and biomimicry across sectors. Given the corporate setting and the above considerations, we hypothesize that the biomimicry LPs tool will increase the creativity [45] of design concepts and the intrinsic motivation of R&D practitioners to apply bio-inspired sustainable design tools to promote sustainable design at the front end of innovation with a significantly lower investment of resources. This outcome would provide further justification for the broader adoption of biomimicry tools in corporate settings with minimal resource expenditure, paving the way for the broader integration of biomimicry in corporate settings to support environmentally sustainable innovation.

## 2. Materials and Methods

### 2.1. Experimental Procedure

R&D professionals from a consumer-packaged goods company were invited to participate in one of two virtual sustainable design tool workshops. Continuing professional development training and workshops such as these are considered activities within the scope of regular job duties, so no extra compensation was provided. Participants were informed of the workshops at their monthly R&D meeting and encouraged to participate via email reminders. They were informed that the two-hour workshop would be conducted virtually via Microsoft Teams and that its focus was to apply a sustainable design tool to redesign a food product. They were randomly assigned to one of two groups, each implementing a different sustainable design tool, and were blind to the study conditions. No prior training in sustainable design was required to participate. A one-hour training tutorial developed by the lead author was circulated a week prior to the workshop to condense the workshops into an appropriate timeframe to ensure feasibility within the corporate setting. The tutorial was a simple introduction to the sustainable design tool to be used and concluded with a case study of tool implementation. A food product was selected due to access to peer-reviewed literature with relevant LCA data and to align with the business category of the company [46]. The workshops were offered one week apart, the first focusing on the application of the biomimicry LPs [28], and the second on the application of the results of an LCA [46]. A simplified LCA report was used to provide a level of content comparable to the biomimicry LPs so that a similar amount of time would be needed by practitioners to consume the content.

Participants volunteered to attend the virtual workshop that best aligned with their own availability. Workshop conditions were consistent across both tools and followed the same timeframe and structure: a one-hour online tutorial circulated the week prior to the workshop, followed by a two-hour online workshop via Microsoft Teams, which was structured as follows:

- 30-min introduction, outlining the session structure and the tool (LCA or Biomimicry LPs, depending on the workshop focus) [28,46]. After a brief discussion about the tutorial, the participants had the opportunity to ask questions. The design objective was then introduced: “Redesign a single-serve yogurt product to improve its environmental sustainability using the provided tool.”
- 15-min group brainstorming, in which participants were divided into small groups (4–5 people) to discuss the design objective and the tool.
- 5-min break.
- 5-min final questions and ideation overview during which the participants had a final opportunity to ask questions about the design objective and tool application. The ideation template, a structured PowerPoint slide, was introduced to guide participants in capturing ratable concepts that included both an illustrative and written concept description and where to select the focus area of each concept (formulation, processing, or packaging).
- 30-min individual ideation, during which the participants received links to one of the sustainable design tools, the biomimicry LPs or the LCA report, along with an ideation template. They worked independently to generate concepts, with facilitators—the main author and a full-time employee—available via Teams chat for assistance.
- 30-min share-out, during which the participants reconvened and all the concepts were compiled into a single PowerPoint. Each participant pitched their top concept in one minute.
- 5-min survey completion, during which the participants were thanked for their participation and directed to complete the online Qualtrics survey.

### 2.2. Participants

A total of 37 R&D personnel participated across two workshops: 19 engaged with the LPs [28], and 18 applied insights from an LCA [46]. Participants were 65% female, 30% male, and 5% unspecified; 38% were aged 18–29, 43% were aged 30–49, and 19% were aged 50–64. Educational backgrounds were highly technical, with 57% holding a master’s degree or higher, and 38% holding a college degree with disciplinary expertise spanning food science, chemistry, and various engineering fields.

### 2.3. Measures

The participants completed a 23-item online Qualtrics survey assessing the practical value and intrinsic motivation to implement either the biomimicry LPs or an LCA report in a product redesign workshop [47,48].

All concepts generated through the workshops that had both visual and written descriptions were evaluated for functional creativity by 13 independent judges, blind to the study conditions, using the Consolidated Creative Solutions Diagnostic Scale. Ratings were collected via Qualtrics over one month. All survey items used a 5-point Likert scale to balance data robustness with time efficiency in a corporate setting [49,50].

#### 2.3.1. Intrinsic Motivation of Tool

Intrinsic motivation was measured via the Intrinsic Motivation Inventory (IMI), a validated 45-item multidimensional scale designed to assess participants’ subjective experiences following task engagement [51]. It comprises six primary subscales: Interest/Enjoyment, Perceived Competence, Effort, Value/Usefulness, Pressure/Tension, and Perceived Choice. A seventh subscale, Relatedness, has been proposed, but remains unvalidated. Due to item redundancy, shorter versions of the IMI are commonly used, and the selection of relevant subscales is standard practice [47,51].

This study focused on two subscales, Interest/Enjoyment and Value/Usefulness, given their relevance to tool engagement and perceived utility. A condensed 9-item version of the IMI was employed. The Interest/Enjoyment subscale, the core indicator of intrinsic motivation, was represented by five items (two reverse-scored). The Value/Usefulness subscale, often used in internalization studies [16] to measure how participants internalize and become self-regulating with respect to activities that they experience as useful or valuable for themselves, contributed four items. As with all self-report instruments, the results should be interpreted with caution, given the modest correlation between self-reported motivation and actual behavior.

#### 2.3.2. Practical Assessment of Tool

To assess the practical value of the sustainable design tools, several criteria were drawn from the literature, particularly a comparative analysis of biomimetic tools [48]. Given the industrial context and comparative nature of the current study, criteria were selected that focused on the practical implementation value of design tools, such as: swiftness (time for implementation), simplicity (tool complexity), and standalone capacity (ability to function independently). Additional criteria, degree of life cycle considered and training specificity, were added to evaluate resource intensity and sustainability. To ensure consistency across groups, we included questions on familiarity with sustainable design practices and the tool used. The survey concluded with questions on interest in applying the tool and open-ended feedback for improvement. While the 9-item scale was not empirically validated, results were analyzed for statistical significance and discussed qualitatively. The survey aimed to assess how R&D employees perceived the tool’s value in environmentally sustainable product redesign.

#### 2.3.3. Creativity of Concepts

The creativity of concepts was measured via the Consolidated Creative Solutions Diagnosis Scale (C-CSDS), a 22-item empirically validated instrument for measuring the functional creativity of product concepts across five categories using domain expert and/or non-expert judges [52,53,54]. The C-CSDS consists of five categories rated along a 5-point Likert scale (1 = not at all, 5 = very much). The categories include (1) Relevance and Effectiveness—the output is fit for purpose, (2) Problematization—the output helps to define the problem/task at hand, (3) Propulsion—the output sheds new light on the problem/task, (4) Elegance—the output is well-executed, and (5) Genesis—the output changes how the problem/task is understood. A final item that rates the Overall Creativity of the concept was included and rated on a five-point Likert scale, creating a final 23-item C-CSDS survey.

The first author recruited 13 judges, both internal and external to the company, who evaluated the creativity of 53 ratable product concepts—those that had both an illustrative and descriptive component—generated during the workshops. Internal judges (n = 4) had expertise in industrial design, sustainability, and product development but did not participate in the study. The nine external judges were Ph.D. and MSc students from the University of Akron. Judges rated the concepts in random order using the 23-item C-CSDS survey via an online Qualtrics platform, blind to the tool condition. The survey was open for one month, with the auto-save feature allowing judges to complete it in multiple sessions.

#### 2.3.4. Focus Area

On the ideation template, the participants were asked to indicate the primary focus area of their concept—Formulation, Processing, or Packaging—to assess which tool better guided attention to the most environmentally impactful component across the product’s life cycle. These categories aligned with the company’s R&D framework. Based on prior LCA data, the first author identified Formulation as the most environmentally detrimental area. Since the LCA report explicitly highlighted this, it was expected that participants using the LCA would focus more heavily on Formulation in their redesign of a single-serve yogurt product.

## 3. Results

**Figure 1:**
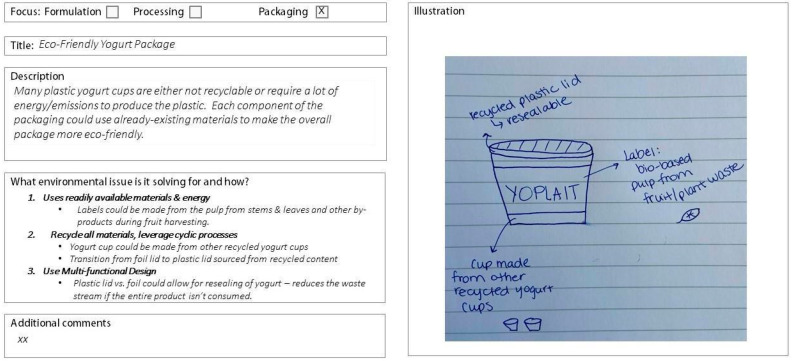
Biomimicry Life Principles concept as created by study participants. Focus categories were removed when rated.

**Figure 2:**
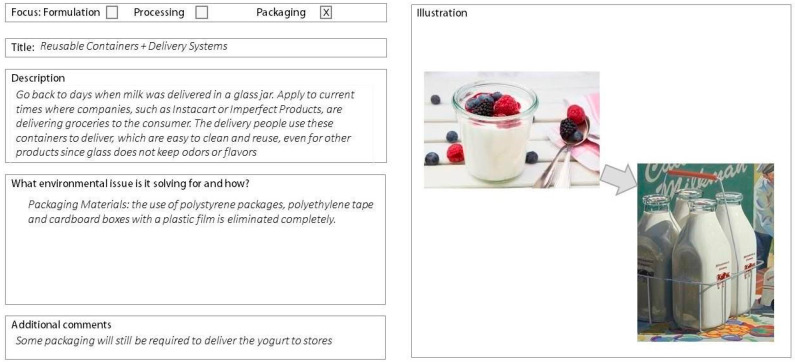
LCA concept as created by study participants. Focus categories were removed when rated.

All 37 workshop participants completed the 23-question survey. 19 participants implemented the biomimicry LPs and 18 used the LCA report results, generating a total of 55 concepts. Two were excluded due to missing descriptions, leaving 53 concepts for analysis—26 from the biomimicry LP group and 27 from the LCA group. Example concepts for LCA and biomimicry LPs are shown in Figure 1 and Figure 2). Please see Table S1 for a summary of all statistical comparisons.

### 3.1. Intrinsic Motivation

The condensed IMI measurement showed a high degree of reliability (Cronbach’s alpha (LCA) = 0.788 and Cronbach’s alpha (biomimicry LPs) = 0.884). Assumptions for normality via the Shapiro–Wilk W test were not met, so the Wilcoxon Ranked Sum test was used to analyze differences across both categories of the theoretical model, which were insignificant (*p* = 0.5690 and *p* = 0.2022, Interest and Enjoyment Factor and Value Usefulness factor, respectively). Similarity of the mean scores (Table 1) demonstrated that both tools were perceived as highly intrinsically motivating.

### 3.2. Creativity

Consistency among the participants was evaluated with Cronbach’s coefficient alpha. Inter-rater reliability was computed for each individual item within the C-CSDS by rotating the dataset to treat the raters as columns and the C-CSDS items as rows [55]. This resulted in a 23 × 651 matrix, resulting in an excellent coefficient alpha of 0.993. ANOVAs were performed on the original five categories of the C-CSDS [55] and Overall Creativity to estimate the effects of judge, tool, and their interaction on the creativity of product concepts (Table 2, Table 3, Table 4, Table 5, Table 6 and Table 7). Judge had 13 levels (13 judges) and tool had two levels (LCA report and biomimicry LPs). A visual inspection of a normal probability plot of the residuals suggests that the data meet the required assumption of ANOVA.

Relevance and Effectiveness Category: An ANOVA showed that there was no significant difference between the tools regarding the Relevance and Effectiveness of concepts generated. There was a significant effect of judge (*p* < 0.0001, partial eta squared = 0.367) but a nonsignificant effect of tool type (*p* = 0.1935) and their interaction (*p* = 0.3015) (Table 2). Compared to LCA, biomimicry LPs did not significantly increase the Relevance and Effectiveness of product concepts generated (3.656 ± 0.039 vs. 3.729 ± 0.039, LCA vs. biomimicry LPs, respectively). Of the 13 judges, 3 rated the concepts generated slightly higher than average (4.673, 4.340, 4.315 ± 0.101), and two judges rated the concepts slightly lower than average (2.786, 2.761 ± 0.101). This category had a weak positive correlation with the overall creativity rating (R = 0.343).

Problematization Category: An ANOVA showed a significant effect of judge (*p* < 0.0001, partial eta squared = 0.148), tool type (*p* = 0.0227, partial eta squared = 0.008) and the interaction between judge and tool (*p* = 0.0449, partial eta squared = 0.045), on the Problematization of product concepts generated (Table 3). Compared to LCA, biomimicry LPs significantly increased the Problematization of product concepts generated (3.095 ± 0.045 vs. 3.243 ± 0.046, LCA vs. biomimicry LPs, respectively). However, four judges gave concepts generated via LCA higher Problematization scores (2.901 vs. 2.638, 4.049 vs. 3.974, 3.235 vs. 3.154, 2.827 vs. 2.692, LCA vs. biomimicry LPs, respectively). Of the 13 judges, 2 rated concepts generally higher than the other judges (3.585 ± 0.117, 4.013 ± 0.117). This category had a strong positive correlation with the Overall Creativity rating (R = 0.625).

Propulsion Category: An ANOVA showed a significant effect of judge (*p* < 0.0001, partial eta squared = 0.248), tool type (*p* < 0.0001, partial eta squared = 0.284) and the interaction between judge and tool (*p* = 0.038, partial eta squared = 0.032) (Table 4). Compared to LCA, biomimicry LPs significantly increased the Propulsion of product concepts generated (2.532 ± 0.041 vs. 2.811 ± 0.042, LCA vs. biomimicry LPs, respectively). However, one judge gave concepts generated by LCA higher Propulsion ratings than biomimicry LPs (2.667 ± 0.150 vs. 2.587 ± 0.153, LCA vs. biomimicry LPs, respectively). Of the 13 judges, two rated concepts generally lower than the remaining judges (1.868 ± 0.107, 1.797 ± 0.107). This category had a strong positive correlation with the overall creativity rating (R = 0.705).

Elegance Category: An ANOVA showed a significant effect of judge (*p* < 0.0001, partial eta squared = 0.228) and tool type (*p* = 0.011, partial eta squared = 0.010). The interaction between judge and tool was nonsignificant (*p* = 0.273) (Table 5). Compared to LCA, biomimicry LPs significantly increased the Elegance of product concepts generated (3.032 ± 0.045 vs. 3.198 ± 0.047, LCA vs. biomimicry LPs, respectively). Of the 13 judges, 2 rated the concepts generally higher than the remaining judges (3.899 ± 0.118, 4.182 ± 0.118). This category had a moderately positive correlation with the Overall Creativity rating (R = 0.548).

Genesis category: An ANOVA showed a significant effect of judge (*p* < 0.0001, partial eta squared = 0.277) and tool type (*p* = 0.0003, partial eta squared = 0.019). The interaction between judge and tool was nonsignificant (*p* = 0.2511) (Table 6). Biomimicry LPs significantly increased the Genesis of product concepts generated compared to the LCA report (2.611 ± 0.039 vs. 2.814 ± 0.040, LCA vs. biomimicry LPs, respectively). Of the 13 judges, 2 rated concepts generally lower than the other judges (1.969 ± 0.101, 1.483 ± 0.101). This category had a strong positive correlation with the overall creativity rating (R = 0.714).

Overall Creativity: An ANOVA showed a significant effect of judge (*p* < 0.0001, partial eta squared = 0.205). The biomimicry LPs tool significantly increased the Overall Creativity of product concepts generated compared to the LCA report (*p* = 0.0010, partial eta squared = 0.016, 2.791 ± 0.047 vs. 3.015 ± 0.048, LCA vs. biomimicry LPs, respectively) (Table 7). Of the 13 judges, 1 judge rated concepts generally lower than the remaining judges (1.868 ± 0.121).

A factor analysis (Oblimin rotation: Refs. [53,55]) was also completed to explore how the data loaded [21]. Due to the limited sample size of the current study, it was important to provide a robust analysis to enable generalizability, which requires a minimum of four items loading on the same factor with a factor loading of at least 0.6 [56]. Thus, these criteria were required when conducting the factor analysis on the Consolidated Creative Solutions Diagnostic Scale. First, factors were considered valid for inclusion with an eigenvalue above 1, and scree plots were developed to confirm factor count by estimating the point of inflection. Second, given the likelihood of correlations between subscales within these measurements, oblique rotation (i.e., direct oblimin) was used for analysis [57].

Finally, the generalizability criteria were confirmed, resulting in the extraction of three dimensions potentially influenced by tool type: (a) Novelty (corresponding to propulsion and genesis), (b) Elegance, and (c) Relevance and Effectiveness, which were consistent with the findings of D. H. Cropley and Kaufman [55] and E.B. Kennedy et al. [21]. The three factors explained 89% of the variance and all had a positive correlation with the Overall Creativity rating ((Novelty = 31%, R = 0.753), (Elegance = 32%, R = 0.517) and (Relevance and Effectiveness = 26%, R = 0.416)). ANOVAs performed on the factor scores demonstrated that, compared to the LCA report, biomimicry LPs significantly increased the Novelty of product concepts generated (*p* < 0.0001, partial eta squared = 0.027, −0.132 ± 0.044 vs. 0.137 ± 0.045, LCA vs. biomimicry LP, respectively) (Table 8) and the Elegance of product concepts generated (*p* = 0.015, partial eta squared = 0.009, −0.093 ± 0.046 vs. 0.065 ± 0.046, LCA vs. biomimicry LPs, respectively) (Table 3, Table 4, Table 5, Table 6, Table 7, Table 8 and Table 9). There was no significant difference between the two tools regarding the Relevance and Effectiveness of product concepts generated (Table 10), yet judges did have a significant effect. Of the 13 judges, three rated the Relevance and Effectiveness of concepts generally higher (0.965 ± 0.105. 0.774 ±0.105, 0.632 ± 0.105) and two judges rated concepts generally lower (−0.958 ± 0.105, −0.955 ± 0.105) than the remaining judges. Of the 13 judges, 2 judges rated the Novelty of concepts lower than the remaining judges (−0.916 ± 0.113, −1.243 ± 0.113), and for the Elegance factor, 2 judges rated concepts generally higher (0.822 ± 0.117, 1.052 ± 0.117) and 1 rated concepts generally lower (−0.674 ± 0.117) than the remaining 10 judges. These findings were quantitatively similar to the ANOVAs completed on the individual categories of the original scale (Table 8, Table 9 and Table 10).

### 3.3. Focus Area

Participants selected a focus area for their concept—Formulation, Processing, or Packaging. From an environmental sustainability standpoint, Formulation was identified as the primary environmental hotspot requiring redesign. Both tools yielded similar proportions of concepts focused solely on Formulation (27% for biomimicry LPs vs. 26% for LCA) and those including it (54% vs. 48%, biomimicry LPs vs. LCA report, respectively).

### 3.4. Practical Value

**Figure 3:**
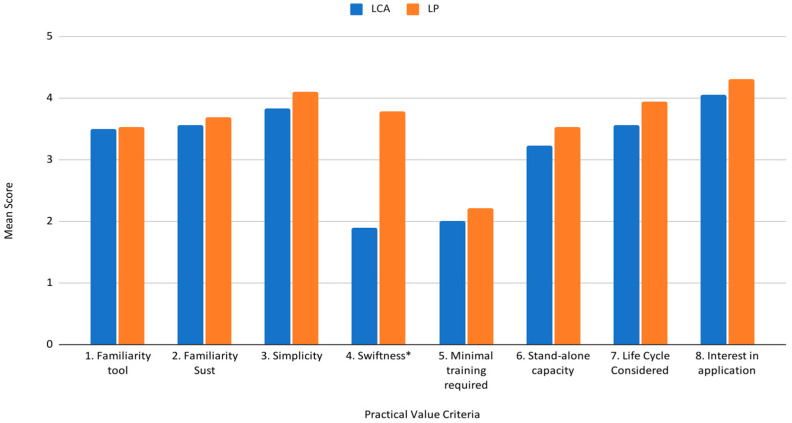
Grouped histogram of the mean score of the practical value assessment criteria (1–8), across both tools. Practical value assessment criteria: 1. Familiarity with tool use, 2. Familiarity with sustainable design, 3. Simplicity, 4. Swiftness, 5. Training specificity required, 6. Stand-alone capacity, 7. Life Cycle Considered, and 8. Interest in application per tool implemented. The key displays the results of LCA in blue and biomimicry LPs in orange. * Statistically significant difference at *p* ≤ 0.0001.

All necessary items were reverse-scored, and the measurement showed a moderate but acceptable degree of reliability (Cronbach’s alpha: 0.6, [58]). The assumption of normality via the Shapiro–Wilk W test was not met, so a Wilcoxon Rank Sums test was used to analyze the results. Figure 3 captures the mean score results across the two tools, LCA report results and biomimicry LPs, for each of the eight criteria of the practical value assessment. The participants within both groups showed equal familiarity with the specific design tool used (criterion 1) and familiarity with sustainable design practices in general (criterion 2), μ = 3.51 and μ = 3.62, respectively. Both tools scored highly across several criteria, such as simplicity (criterion 3), stand-alone capacity (criterion 6), degree of life cycle considered (criterion 7), and the overall interest in applying the tool to a project in their own work (criterion 8), with mean values ranging from 3.38 to 4.19. Both tools scored low for criterion 5, the degree of specific training required, indicating that both tools would require some degree of specific training for implementation, μ = 2.11. Criterion 4, swiftness, the necessary time for the implementation of the tool, was the only practical criterion that was significantly different between the tools. Compared to LCA, biomimicry LPs significantly decreased the time necessary for implementation (μ = 1.88, std = 0.963, Mdn = 2 vs. μ = 3.79, std = 1.031, Mdn = 4, *p* ≤ 0.0001 *, LCA vs. biomimicry LPs, respectively).

## 4. Discussion

In our study, both the biomimicry LPs and LCA yielded concepts of similar relevance and effectiveness, scoring highest in this category of creativity (C-CSDS), with a similar number of concepts solely focused on “Formulation,” identified as the environmental hotspot in need of redesign. This result provides a strong practical justification for biomimicry LPs as an alternative tool to LCA. In addition, the biomimicry LPs generated solutions that were significantly more novel and elegant, aligning with earlier findings highlighting biomimicry’s potential to increase ideation quality at the front end of innovation [21,31]. Further analysis showed that biomimicry LPs outperformed LCA in four of the five categories of creativity (C-CSDS)—Propulsion, Genesis, Problematization, and Elegance. The Propulsion and Genesis categories (loaded together as the Novelty factor) examine situational and future novelty, respectively. Situational novelty suggests that a solution is original and offers a new perspective on the problem/task at hand, while future novelty goes beyond the problem/task at hand to conceptualize a broader range of issues in a novel way. Elegance is defined as a solution that strikes the rater as beautiful, refined, and harmonious [55]. The combination of these three categories is important for a product to be successful in the market because novelty and elegance increase consumers’ interest in the product itself and their willingness to pay for it [59]. Finally, the Problematization category rates solutions according to how well they draw attention to problems relative to what already exists [55]. The biomimicry LPs significantly outperformed the LCA tool in the Problematization category, demonstrating their potential to build awareness of overlooked issues within a product category. This ability to draw attention to challenges within existing solutions represents a valuable contribution to early-stage innovation and long-term impact. Practitioners’ open comments added to this finding, reporting that the “Biomimicry LPs tool could provide the basis for needed conversation with regulatory entities regarding current supply chain challenges and how to tackle the effective deployment of solutions for long-term projects,” whereas comments from LCA participants were focused more on the potential of the tool to “enable manufacturers to get the most ‘bang-for-the-buck’”. These comments provided insight into the types of mindset that these two tools evoke in participants, with the biomimicry LPs promoting a more whole-systems thinking mindset compared to the narrow-focus mindset promoted through the LCA tool.

Against this backdrop, it is noteworthy that the biomimicry LPs—a generative, low-resource tool—elicited marginally higher levels of intrinsic motivation across both the Interest/Enjoyment and the Value/Usefulness subscales. This finding was supported by open comments provided by the study participants who implemented the LCA report, who commented, “Although an LCA could really impact our business, a lot of time and dedicated specialized resources would be required, thus a barrier to implementation,” and “I think you could give an example of a more qualitative approach and people might see it as more immediately applicable to their work.” This outcome is particularly promising given initial concerns that a qualitative tool might not resonate with a technically oriented audience. Moreover, biomimicry LPs generated concepts of comparable relevance, but also resulted in significantly more novel and elegant solutions while fostering a systems-thinking mindset. These findings suggest that biomimicry LPs provide a viable pathway for embedding sustainability into early-stage innovation by offering a compelling combination of creative enhancement, cognitive engagement, motivational appeal, and practical feasibility. This was qualitatively supported in the current study via the practical assessment survey; however, this survey instrument was not quantitatively validated at the time of the study. Therefore, caution is advised when interpreting these results, as future research using a validated tool is required to draw firm conclusions.

With that said, both tools scored highly across most categories of practical value, suggesting that both tools were perceived as useful at the front end of a product redesign project for environmental sustainability. Both tools were easy to use, even in a standalone capacity, and both considered a high degree of the life cycle of the product being redesigned. Both tools were perceived as requiring a degree of specific training; however, in the current study, practitioners were provided with only a one-hour training tutorial prior to the two-hour virtual workshop. Thus, one could argue that only minimal training is required for R&D practitioners to explore these tools. Finally, implementing the results of the LCA showed that it would require a substantial investment of time compared to the biomimicry LPs. Time is a constant constraint in a corporate R&D setting [60], so the more streamlined the front end of the innovation process, the better. The participants’ open comments supported this, stating that “full implementation of an LCA would be too intensive, but implementing a basic report upfront as much as possible could act as a simplistic way for an innovation session, utilizing the mindset to identify better ways ‘in’ before fully defining a project”.

## 5. Conclusions

The results of this study underscore the value of providing corporate practitioners with flexible, accessible tools that can be seamlessly integrated into the design process, lowering adoption barriers and increasing the likelihood of routine use [34]. The biomimicry LPs, as demonstrated here, offer a promising entry point for integrating biomimicry into corporate innovation practices. Functioning as a standalone, easy-to-use tool, the biomimicry LPs require minimal training and no specialized biological knowledge, yet they generate concept outcomes comparable in relevance to those produced using traditional tools like LCA. Moreover, biomimicry LPs generated concepts that exhibited marked improvements in novelty and elegance, required less time to implement, and fostered systems thinking—an essential mindset for sustainable design. These findings present a compelling case for both practitioners and corporate leadership to adopt the biomimicry LPs as a strategic addition to an existing toolkit of sustainable design tools. While previous case studies, such as Kennedy and Marting [31], demonstrated the potential of biomimicry design, following extensive biological analysis and expert input, the approach introduced here significantly reduces the resource burden. By eliminating the need for biological model selection and abstraction, the biomimicry LPs streamline the integration of biomimetic thinking into real-world workflows. As such, they represent a pragmatic and scalable pathway to catalyze broader and deeper engagement with biomimicry across corporate contexts—laying the foundation for more innovative, efficient, and ecologically aligned product development.

The findings of our study demonstrate the significant potential of biomimicry LPs as catalysts for sustainable design thinking, offering compelling evidence to support their integration into corporate innovation strategies. By highlighting their efficacy and accessibility, the results underscore the viability of biomimicry-based approaches as low-resource, high-impact tools—strengthening the case for their widespread adoption in advancing environmentally sustainable innovation within corporate environments.

### Future Work and Limitations

There were several limitations to the current study, and future work is required to build on these results. First, an increased sample size across a variety of industries is warranted to determine the generalizability of the results found. COVID-19 restrictions required this study to be conducted completely virtually, so it would be valuable to conduct this study in person, given that in-person ideation is the traditional practice within an industrial setting. Also, the self-reporting nature of the survey would benefit from additional research gathering data that correlates to actual behavior. Additionally, an empirically validated practical use assessment tool to confirm the results found in the present study and to enable a comparison of the tools analyzed in the comparative study of biomimetic tools [48] would be beneficial. Finally, given the nature of this study, being conducted at the front end of the innovation process, quantitative analysis regarding the environmental sustainability of the concepts was not possible on the concepts generated. An open and important research question involves how the results discussed here for concept generation translate into actual product development.

## Figures and Tables

**Table 1:** Mean scores for the Intrinsic Motivation inventory subscales.

	Interest/Enjoyment	Value/Usefulness
LCA	4.3	3.4
Biomimicry LPs	4.4	3.6

**Table 2:** ANOVA, effects on the Relevance and Effectiveness of product concepts generated.

Relevance and Effectiveness				
Sources	DF	SS	F Ratio	*p*
Judge	12	208.001	32.119	<0.0001 *
Tool	1	0.914	1.694	0.194
Judge*Tool	12	7.571	1.169	0.302

* Significant effect.

**Table 3:** ANOVA, effects on the Problematization of product concepts generated.

Problematization				
Sources	DF	SS	F Ratio	*p*
Judge	12	83.088	9.592	<0.0001 *
Tool	1	3.764	5.213	0.023 *
Judge*Tool	12	15.579	1.798	0.045 *

* Significant effect.

**Table 4:** ANOVA, effects on the Propulsion of product concepts generated.

Propulsion				
Sources	DF	SS	F Ratio	*p*
Judge	12	133.579	18.25	<0.0001 *
Tool	1	13.38	21.935	<0.0001 *
Judge*Tool	12	13.515	1.847	0.038 *

* Significant effect.

**Table 5:** ANOVA, effects on the Elegance of product concepts generated.

Elegance				
Sources	DF	SS	F Ratio	*p*
Judge	12	143.695	16.306	<0.0001 *
Tool	1	4.761	6.483	0.011 *
Judge*Tool	12	10.645	1.208	0.273

* Significant effect.

**Table 6:** ANOVA, effects on the Genesis of product concepts generated.

			Genesis	
Sources	DF	SS	F Ratio	*p*
Judge	12	138.392	21.175	<0.0001 *
Tool	1	7.117	13.067	0.0003 *
Judge*Tool	12	8.104	1.24	0.251

* Significant effect.

**Table 7:** ANOVA, effects on the Overall Creativity of product concepts generated.

Overall Creativity				
Sources	DF	SS	F Ratio	*p*
Judge	12	129.574	14.036	<0.0001 *
Tool	1	8.445	10.97	0.001 *
Judge*Tool	12	11.54	1.249	0.245

* Significant effect.

**Table 8:** ANOVA, effects on the Novelty of product concepts generated.

			Novelty	
Sources	DF	SS	F Ratio	*p*
Judge	12	170.316	20.729	<0.0001 *
Tool	1	12.537	18.309	<0.0001 *
Judge*Tool	12	13.501	1.643	0.0755

* Significant effect.

**Table 9:** ANOVA, effects on the Elegance of product concepts generated.

Elegance				
Sources	DF	SS	F Ratio	*p*
Judge	12	151.515	17.375	<0.0001 *
Tool	1	4.301	5.918	0.0153 *
Judge*Tool	12	10.133	1.162	0.3070

* Significant effect.

**Table 10:** ANOVA, effects on the Relevance and Effectiveness of product concepts generated.

Relevance & Effectiveness				
Sources	DF	SS	F Ratio	*p*
Judge	12	227.460	32.628	<0.0001 *
Tool	1	1.471	2.5330	0.1120
Judge*Tool	12	7.846	1.126	0.3352

* Significant effect.

## Data Availability

Data available upon request from the authors.

## Supplementary Materials

The following supporting information can be downloaded at: https://www.mdpi.com/article/10.3390/biomimetics10100667/s1. Table S1: “A” shows the number of unique participants in the study and the concepts generated. Formulation focus denotes whether the participants focused exclusively on product formulation in their LCA/LP design or included packaging and/or other aspects of the product. “B” lists results of statistical comparisons of outcomes with respect to the survey tool specified in MS.
