# Supplementary material for: A Comparative Analysis of Sustainable Design Tools for Product Redesign Within a Business Context
Source: Biomimetics (Basel). 2025 Oct 3;10(10):667. doi: 10.3390/biomimetics10100667 (PMC12561651; doi:10.3390/biomimetics10100667)
Supplement: Supplementary file 1 [file biomimetics-10-00667-s001.zip › biomimetics-3803400-supplementary.pdf]

Supplementary Table S1. "A" shows the number of unique participants in the study and the concepts generated. Formulation focus denotes whether the participants focused exclusively on product formulation in their LCA/LP design or included packaging and/or other aspects of the product. "B" lists results of statistical comparisons of outcomes with respect to the survey tool specified in MS.

			Formulation Focus	
**A. Number of participants**	**Design Tool Used**	**Number of Concepts**	**Solely**	**Included**
19	LP	26	27%	54%
18	LCA	27	26%	48%

**B. Survey Tool**	**Question Focus**	**Comparative Result**
**C-SDS**	* Relevance & Effectiveness	No significant difference
	*Novelty (Propulsion & Genesis)	LPs significantly increased
	• Genesis	LPs significantly increased
	• Problemization	LPs significantly increased
	Propulsion	LPs significantly increased
	*Elegance	LPs significantly increased
	• Overall creativity	LPs significantly increased
**Practical Use**	1. Familiarity with tool use	No significant difference
	2. Familiarity with Sustainable design	No significant difference
	3. Simplicity	No significant difference
	4. Swiftness	LPs significantly increased
	5. Training specificity required	No significant difference
	6. Stand-alone capacity	No significant difference
	7. Life Cycle Considered	No significant difference
	8. Interest in application per tool implemented.	No significant difference
**IMI**	Interest/Enjoyment	No significant difference
	Value/Usefulness	No significant difference
